# Short-Term Effect of Induced Alterations in Testosterone Levels on Fasting Plasma Amino Acid Levels in Healthy Young Men

**DOI:** 10.3390/life11111276

**Published:** 2021-11-22

**Authors:** K. Barbara Sahlin, Indira Pla, Jéssica de Siqueira Guedes, Krzysztof Pawłowski, Roger Appelqvist, György Marko-Varga, Gilberto Barbosa Domont, Fábio César Sousa Nogueira, Aleksander Giwercman, Aniel Sanchez, Johan Malm

**Affiliations:** 1 Section for Clinical Chemistry, Department of Translational Medicine, Lund University, Skåne University Hospital Malmö, 205 02 Malmö, Sweden; indira.pla_parada@med.lu.se (I.P.); krzysztof_pawlowski@sggw.edu.pl (K.P.); roger.appelqvist@bme.lth.se (R.A.); gyorgy.marko-varga@bme.lth.se (G.M.-V.); johan.malm@med.lu.se (J.M.); 2 Clinical Protein Science & Imaging, Biomedical Centre, Department of Biomedical Engineering, Lund University, BMC D13, 221 84 Lund, Sweden; 3 Laboratory of Proteomics, LADETEC, Institute of Chemistry, Federal University of Rio de Janeiro, Rio de Janeiro 21941-909, Brazil; guedesjessica@outlook.com (J.d.S.G.); fabiocsn@iq.ufrj.br (F.C.S.N.); 4 Proteomics Unit, Institute of Chemistry, Federal University of Rio de Janeiro, Rio de Janeiro 21941-909, Brazil; gilberto@iq.ufrj.br; 5 Department of Molecular Biology, University of Texas Southwestern Medical Center, Dallas, TX 75390, USA; 6 Department of Biochemistry and Microbiology, Institute of Biology, Warsaw University of Life Sciences SGGW, 02707 Warszawa, Poland; 7 First Department of Surgery, Tokyo Medical University, 6-7-1 Nishishinjiku, Shinjiku-ku, Tokyo 1600023, Japan; 8 Molecular Reproductive Medicine, Department of Translational Medicine, Lund University, 205 02 Malmö, Sweden; aleksander.giwercman@med.lu.se

**Keywords:** testosterone, protein breakdown, gluconeogenesis

## Abstract

Long term effect of testosterone (T) deficiency impairs metabolism and is associated with muscle degradation and metabolic disease. The association seems to have a bidirectional nature and is not well understood. The present study aims to investigate the early and unidirectional metabolic effect of induced T changes by measuring fasting amino acid (AA) levels in a human model, in which short-term T alterations were induced. We designed a human model of 30 healthy young males with pharmacologically induced T changes, which resulted in three time points for blood collection: (A) baseline, (B) low T (3 weeks post administration of gonadotropin releasing hormone antagonist) and (C) restored T (2 weeks after injection of T undecanoate). The influence of T on AAs was analyzed by spectrophotometry on plasma samples. Levels of 9 out of 23 AAs, of which 7 were essential AAs, were significantly increased at low T and are restored upon T supplementation. Levels of tyrosine and phenylalanine were most strongly associated to T changes. Short-term effect of T changes suggests an increased protein breakdown that is restored upon T supplementation. Fasting AA levels are able to monitor the early metabolic changes induced by the T fluctuations.

## 1. Introduction

In healthy males, catabolic (tissue breakdown) and anabolic (tissue repair) processes are in balance throughout the diurnal cycle to maintain muscle mass [1]. Skeletal muscle is the main source of protein for catabolism during energy shortage, caused by fasting or exercise [2]. Gluconeogenesis can provide energy in catabolic states utilizing amino acids (AAs). 

Because essential AAs cannot be endogenously synthesized, they are good tracers of protein turnover, which is the synthesis and breakdown of proteins [3,4]. Particularly, Branched Chain Amino Acids (BCAAs) and aromatic AAs have been extensively studied [5,6,7]. BCAAs include isoleucine (Ile), leucine (Leu) and valine (Val), and are preferentially degraded in muscle tissue [8]. The aromatic AAs include tryptophan (Trp), tyrosine (Tyr) and phenylalanine (Phe), of which Phe and Tyr cannot be synthesized or degraded in muscles. They can only be degraded in the liver meaning that Phe and Tyr can be used to monitor the net rate of protein degradation [8].

Testosterone (T) has a highly complex metabolic effect on the major tissues involved in insulin action, such as the liver, fat and muscle tissues [2,9,10]. Research suggests that long-term T deficiency causes metabolic changes, such as favoring net protein breakdown resulting in decreased muscle mass. Short- and long-term effects of T supplementation suggest an increased rate of protein synthesis [1]. T has been shown to increase glucose transport via upregulation of GLUT4 expression, facilitating insulin signaling by increasing expression of insulin receptor substrate 1 and 2, and increasing glycogen synthesis by raising the activity of glycogen synthase in skeletal muscle. Additionally, increased adipose mass, reduced insulin sensitivity, impaired glucose tolerance, elevated triglycerides and cholesterol, and low HDL-cholesterol are all symptoms of metabolic syndrome and are all also associated with T deficiency [10]. 

Previously, we have designed a human model with 30 healthy young males, with induced abrupt T fluctuations without interference of T related comorbidities. The model yields plasma from three time points: baseline, low T and restored T [11]. This enables us to study the possible T effects on protein turnover and the causality in development of comorbidities. Thus, monitoring fasting plasma AA levels in response to changes in T levels in the healthy human model could provide evidence to the early unidirectional effects of T changes before the appearance of comorbidities and clues to the pathogenesis and consequences of T deficiency. We hypothesize that metabolic changes occur with short-term T alterations and therefore aim to detect a change in fasting plasma AA levels in healthy young men with pharmacologically induced alterations in T levels.

## 2. Materials and Methods

### 2.1. Subjects and Study Outline

Thirty healthy male volunteers of 23.9 years (19–32 years) with BMI 23 (19.1–26.9 kg/m^2^), were subjected to T deprivation by subcutaneous administration of 240 mg of gonadotropin releasing hormone antagonist (Degaralix^®^, Ferring Pharmaceuticals). After 3 weeks, 1000 mg of T undecanoate (Nebido^®^, Bayer Pharmaceuticals) were given intramuscularly to restore T levels. Blood was collected at three-time points (A) baseline, (B) low T (3 weeks after Degaralix^®^) and (C) restored T (2 weeks after Nebido^®^). 

**Figure 1:**
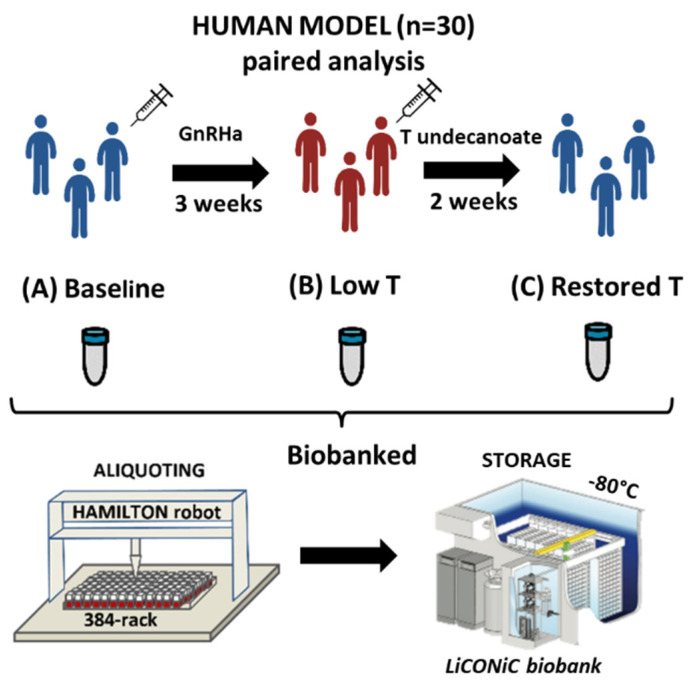
An overview of the pharmacologically induced testosterone (T) states with three time points: (A) baseline (B) Low T (due to chemical castration) and (C) Restored T. Blood collected from each time point was aliquoted and stored in −80 °C prior to analysis within a cycle time of two hours.

The study was approved by the Swedish Ethical Review Authority (Approval number: DNR 2014/311, date of approval 8 May 2014). Inclusion criteria were 19–32 years of age, healthy, BMI 20–25 kg/m^2^, non-smoker or occasional smoker. Exclusion criteria included regular medication, exposure to anabolic steroids, drugs of abuse used within the last year or ever presenting with a stroke, heart and liver disease, cancer or any other chronic disease [11]. The samples were collected from subjects in fasting condition, aliquoted and stored at −80 °C. The cycle time from sample collection to storage was below two hours to maintain sample integrity [12]. For an overview of the model and samples handling, see Figure 1. Thirty-eight well-known biomarkers were previously reported, including time point measurements of T, follicular stimulating hormone and luteinizing hormone [11]. The concentrations of the reproductive hormones at the three time points can be viewed in Table 1.

### 2.2. Amino Acid Analysis

The plasma collected from the fasting healthy subjects was analyzed at the Clinical Chemistry Laboratory at Skåne university hospital in Malmö, Sweden. One sample was not available in the biobank and was not included for further analysis. Briefly, internal standards were added to the plasma samples after which the proteins were precipitated with sulfosalicylic acid and the pH was adjusted with lithium hydroxide. The free AAs were analyzed automatically on a Biochrome 30+ AA analyzer (Biochrom Ltd., Cambridge, England). The ninhydrin complexes of the individual AAs were quantified by spectrophotometry at 570 and 440 nm. The AAs were identified by their retention times. The concentration was automatically calculated by the integration program. The AA standards used were basic (A6282 Sigma-Aldrich, Darmstadt, Germany) and acidic and neutral (A6407 Sigma-Aldrich) as well as L-glutamine (56-85-9 Sigma-Aldrich, Darmstadt, Germany). Twenty-three AAs were measured in the protocol: alanine (Ala), α-aminobutyrate (Aaba), arginine (Arg), asparagine (Asn), aspartate (Asp), citrulline (Citr), cysteine (Cys), glutamine (Glu), glycine (Gly), histidine (His), Ile, Leu, lysine (Lys), methionine (Met), 3-methylhistidine (3-Mhis), ornithine (Orn), Phe, serine (Ser), taurine (Taur) threonine (Thr), Trp, Tyr, and Val. Confidence intervals for the measurements can be found in the supplementary material Table S1: (Low, high, SD and CI for the amino acids).

### 2.3. Statistical Analysis

#### 2.3.1. Monitoring Free Amino Acids across Time Points with Testosterone Fluctuations

Concentrations of AAs were first log2 transformed in order to attempt a normal distribution. Time points were compared by one-way paired ANOVA test (R function: ezANOVA{ez}) for the AAs with normal distribution. For the AAs with non-normal distribution the non-parametric Friedman rank sum test was applied. The list of *p*-values obtained for each AA was adjusted (*q*-value) to control the false discovery rate (FDR) (‘fdr’ method). To detect specific statistically significant changes between time points the ANOVA and Friedman rank sum tests were followed by post hoc pairwise tests based on paired Student’s *t*-test (two-tails) and Wilcoxon rank test respectively for 29 participants. *P*-values obtained from pairwise analyses were adjusted to control Type I errors from multiple comparisons, in which adjusted (FDR) *p*-values < 0.05 were considered significant. As a complementary analysis, we used the 95% confidence intervals (CIs) [13] of the mean of the differences between time points to confirm previous analyses. The change was considered to be statistically significant if the CI did not include zero.

#### 2.3.2. Modeling the Best Amino Acids in Response to Testosterone Fluctuations

To select the best AAs that reflect the effects of T changes on fasting plasma AA levels, we performed a stepwise-binomial logistic regression (method: backward). We tested all possible combinations of AAs influenced by T changes to discriminate group B (low T) from group A and C (baseline and restored T). A Bootstrap resampling with replacement method was applied to assess the consistency of the predictors selected from the stepwise-binomial logistic regression (*p*-value < = 0.05 for acceptance). The model was derived from the predicted log-odds (of being low T) obtained from a binomial logistic regression analysis, in which the dichotomized variable ‘testosterone level’ (0: normal T (time points A, C) and 1: low T (time point B)) was used as the dependent variable and the expression of the selected AAs from the stepwise regression were the variables of the predictors. All analyses described in this part were performed in R software (stepAIC {MASS}; boot.stepAIC {bootStepAIC}).

## 3. Results

Fifteen out of 23 AAs had significant overall changes represented by *q*-values less than 0.05: Asn, Asp, Arg, Cys, Glu, His, Leu, Lys, Met, Phe, Ser, Taur, Trp, Tyr, and Val (Table S2: All statistical results). The change in reproductive hormones can be viewed in Table 1. T exhibits a significant difference between B-A and C-B, whereas luteinizing hormone and follicular stimulating hormone only change significantly between A-B and remain at low levels in both time point B and C.

### 3.1. Amino Acids Significantly Changed by Testosterone

Nine out of 15 AAs that had significant overall changes were specifically affected by T changes, because they significantly increased with low T and restored upon T supplementation. These AAs include Asn, Val, Met, Leu, Tyr, Phe, Lys, His, and Trp (Table 2). Seven out of nine AAs that significantly changed with T were essential AAs, meaning that all but two essential AAs changed (i.e., Ile and Thr). Tyr and Asn were the only two non-essential AAs that were significantly affected by T. All changes were corroborated by the analysis of confidence intervals of the means, in which none of them included zero.

**Figure 2:**
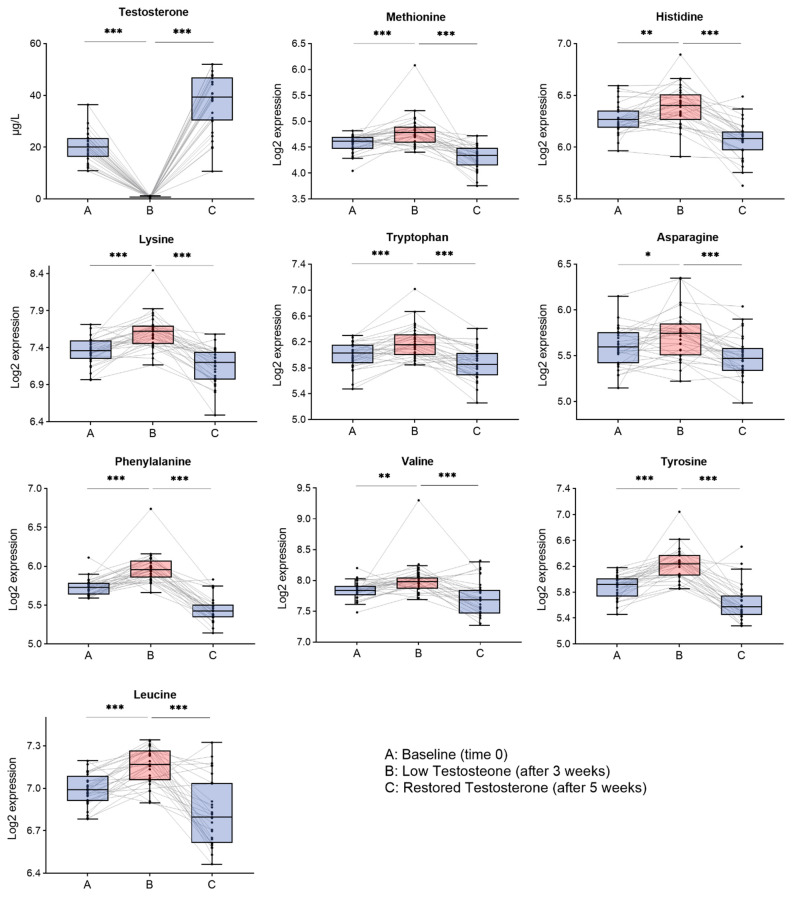
Box-plots distribution at (A) baseline, (B) low T and (C) restored T for nine AAs that have at least significant change in B-A and C-B (FDR < 0.05). The AAs are negatively influenced by T: aspargine (Asn), histidine (His), leucine (Leu), lysine (Lys), methionine (Met), phenylalanine (Phe), tryptophan (Trp), tyrosine (Tyr), and valine (Val), meaning that they are increased with low T and restored upon T supplementation. * *p* < 0.05, ** *p* < 0.01, *** *p* < 0.001.

The box-plot distribution of the intensities across the time points is shown in Figure 2, as well as the changes in the individual subjects (represented by each line in the figure). Levels of all AAs were negatively associated with those of T (Asn, His, Leu, Lys, Met, Phe, Trp, Tyr, and Val).

### 3.2. Branched Chain Amino Acids and Aromatic Amino Acids Significantly Changed by Testosterone

Leu (B-A *p* < 0.001, C-B *p* < 0.0001) and Val (B-A *p* < 0.01, C-B *p* < 0.001) were the BCAAs that changed significantly with T changes (Table 2). Tyr (B-A *p* < 0.0001, C-B *p* < 0.0001), Trp (B-A *p* < 0.001, C-B *p* < 0.0001) and Phe (B-A *p* < 0.0001, C-B *p* < 0.0001) significantly change with T. Ile was the only BCAA that does not change significantly with T (B-A *p* < 0.340, C-B *p* < 0.390) (Table S2).

### 3.3. Selected Amino Acids That Best Reflect Changes in Testosterone

The combination of AA levels that best explain the T changes contains Phe and Tyr, which were selected as significant (*p* < = 0.05) 77 % and 50 % of times respectively (by bootstrap resampling procedure) and it was represented by the linear combination: Time_point = −95.4 + 3.44 Tyr + 12.76 Phe (see Supplementary Results: stepwise-binomial logistic regression and Bootstrap resampling).

## 4. Discussion

Our results clearly suggest an early anabolic effect of T on protein turnover, which can be measured by changes in fasting plasma AA composition. Seven out of nine AAs that significantly change with T were essential AAs, such as Phe. Essential AAs are considered good tracers of protein turnover, because essential AAs are released to produce energy in the catabolic state [4]. Additionally, Tyr is sometimes considered to be an essential AA as it is synthesized by irreversible hydroxylation of Phe in the liver. Flux of Phe levels provides a good representation of whole-body protein breakdown [14,15]. We found that Phe and Tyr, when combined, better reflect the T effect in the human model. 

Previously in the human model (*n* = 30), alanine aminotransferase and aspartate aminotransferase, enzymes involved in AA metabolism, and urea, a waste product of protein breakdown, all changed suggesting that low T induces increased protein breakdown [11]. Furthermore, unbiased proteomic analysis of the same human model yielded that gluconeogenesis was upregulated during low T, suggesting that low T favored a catabolic state [16]. The most common source of protein in the fasting state is muscle tissue [2], therefore the increase in liver markers that are involved in gluconeogenesis suggests an increased muscle degradation [17]. As this is found in a healthy human model, the causality of increased AA levels is clear, because the results are not impacted by confounding factors, such as comorbidities. 

To our knowledge, this is the first time that AAs have been studied in relation to early anabolic effects with induced low T in healthy males with subsequent restoration with supplemented T. The only other study measuring AAs on healthy subjects, compared baseline with injected T in seven young healthy males in a five-day interval, and found a net increase in protein synthesis after injection [18]. Our results support our previous findings in the human model and suggest that an increase of specific AA levels could indicate T deficiency and, thereby, enable possible implementation of preventive measures prior to the onset of metabolic comorbidities, such as insulin resistance and type II diabetes mellitus (DM2). In addition, several studies suggest that there is a positive influence of insulin resistance on BCAAs [5,6,7], and some evidence suggests that the influence is causal. A longitudinal study on BCAAs and aromatic AAs found that the combination of Ile, Tyr and Phe gave a five-fold higher risk of future development of DM2 [19]. 

Most of the AA changes in the present study were observed in previous studies on hypogonadism or T related disease as well as DM2 [9,19,20]. Notably, Tyr was reported significantly up regulated in hypogonadal males versus controls, while both Tyr and Phe were among the best risk predictors of developing DM2 [19]. In contrast, Asn, a non-essential AA, seemed to reflect the protein breakdown process in the present study. Interestingly, a previous study comparing diabetic obese men with non-diabetic obese men, found that fasting insulin levels is also related to Asn levels [20].

A metabolomic study comparing hypogonadal men with matched controls for age and BMI, found that, similar to our study, Tyr and Lys were negatively associated with T levels. In contrast, they also found that Ala, Arg and Ser were negatively associated with T [9], which was not found in the present study. A factor that could account for this difference is that the subjects in the present study were healthy and were their own controls, which was not the case in the study by Fanelli et al. 

Even though our human model is limited to 30 subjects, it is a considerably larger number of subjects than most other studies found, which often include under 15 subjects. A limitation of our study is that potential confounders that affect plasma AA composition were not standardized, such as subject diet and exercise. However, the blood was drawn in overnight fasting condition. A study that investigated the variation of AAs in healthy males with different protein intake and exercise levels, hypothesized that the AAs, especially essential AAs, would be more variable in the fed state and less variable in fasting state. Contrarily, the results suggest that Tyr, Met and Leu were the AA markers least affected by protein intake in the fasting state, while fasting Phe was lower in the high protein group along with fasting Ser, Ile, and Val [21]. 

On the other hand, there is a documented effect of exercise on AA concentration in plasma [8,22,23,24,25,26,27]. In general, the change in AA levels depended on the type of exercise training and individual performance, comparing rest vs exercise time points. The individual AA changes that vary across studies and comparisons were based on the changes before and after exercise, with an overnight fasting state as the baseline or control. However, when comparisons were reported in a longitudinal five-week experiment that involved 11 competitive athletes with fasting overnight AA levels before and after training, most AAs decreased in plasma levels after the training period, except for Lys, Ser, Asn, Asp, Tyr and Phe [25].

Taking all together, the studies suggest that plasma AAs are reliable markers over time to predict metabolic shifts, especially when measured in overnight fasting. Some AAs seem to be better at withstanding certain differences in lifestyle, which can be difficult to control in a clinical setting. Additionally, some of the AAs in the present study were found to have a clear association with long term T deficiency and previous studies found a similar link between AAs and metabolic diseases. Consequently, this analysis supports the evaluation of T driven influence on AA levels, particularly Phe and Tyr. These may serve as possible early prognostic markers in the development metabolic comorbidities provoked by T deficiency, such as insulin resistance, DM2 and metabolic syndrome. However, further studies are needed.

In conclusion, we report that fasting plasma AA levels are influenced by T. Low T levels mainly increase levels of essential AAs, suggesting protein breakdown, which can be restored by T supplementation. The combination of Tyr and Phe yield the best results to monitor T changes in our human model.

## Figures and Tables

**Table 1:** Basic parameters of 30 healthy male subjects at different time points of the model * showing mean and standard deviation: (A) baseline, (B) low T and (C) restored T. T was low in B and high in C; whereas, follicular stimulating hormone (FSH) and luteinizing hormone (LH) were low in both B and C.

Parameter	Baseline (A)	Low T (B)	Restored T (C)
T (nmol/L)	19.97 ± 5.7	0.71 ± 0.3	37.39 ± 11.1
FSH (IU/L)	3.09 ± 1.7	0.15 ± 0.1	0.14 ± 0.1
LH (IU/L	4.99 ± 1.6	0.14 ± 0.1	0.14 ± 0.1

* Results are from our previous publication on the human model [11].

**Table 2:** Significant AAs are shown that have at least significant p-values in B-A and C-B, with confidence intervals of differences in means, and p-values with time point comparisons.

AA	*q*-Value (FDR < 0.05)	CI (B-A)	*p*-Value (B-A)	CI (C-B)	*p*-Value (C-B)	CI (C-A)	*p*-Value (C-A)
Asn	<0.001 ^a^	0.117	<0.05	−0.229	<0.0001	−0.112	<0.05
		(0.011; 0.222)		(−0.34; −0.119)		(−0.198; −0.027)	
Val ^e^	<0.001 ^b^	0.159	<0.01	−0.323	<0.001	−0.146	<0.05
		(0.073; 0.23)		(−0.47; −0.187)		(−0.278; −0.015)	
Met ^e^	<0.0001 ^b^	0.215	<0.001	−0.448	<0.001	−0.253	<0.01
		(0.123; 0.314)		(−0.589; −0.321)		(−0.379; −0.082)	
Leu ^e^	<0.0001 ^b^	0.191	<0.001	−0.376	<0.0001	−0.191	<0.01
		(0.136; 0.24)		(−0.508; −0.264)		(−0.286; −0.086)	
Tyr	<0.0001 ^b^	0.286	<0.0001	−0.615	<0.0001	−0.281	<0.001
		(0.211; 0.413)		(−0.737; −0.492)		(−0.393; −0.18)	
Phe ^e^	<0.0001 ^b^	0.249	<0.0001	−0.547	<0.0001	−0.291	<0.0001
		(0.196; 0.303)		(−0.623; −0.473)		(−0.363; −0.223)	
Lys ^e^	<0.0001 ^b^	0.245	<0.0001	−0.431	<0.0001	−0.214	<0.01
		(0.171; 0.319)		(−0.567; −0.335)		(−0.31; −0.097)	
His ^e^	<0.0001 ^a^	0.117	<0.01	−0.324	<0.0001	−0.207	<0.0001
		(0.038; 0.195)		(−0.405; −0.243)		(−0.277; −0.138)	
Trp ^e^	<0.0001 ^b^	0.205	<0.001	−0.338	<0.0001	−0.129	<0.05
		(0.117; 0.302)		(−0.423; −0.226)		(−0.238; −0.017)	
T	<0.0001 ^a^	−19.262	<0.0001	36.683	<0.0001	17.421	<0.0001
		(−21.45; −17.075)		(32.451; 40.914)		(13.004; 21.837)	

^e^ = essential amino acid, ^a^ = ANOVA test, with adjacent paired *t*-test for time point comparisons, ^b^ = Freidmans test, with adjacent paired Wilcoxon test for time point comparisons.

## Data Availability

Data is contained within the article or supplementary material.

## Supplementary Materials

The following are available online at https://www.mdpi.com/article/10.3390/life11111276/s1, Table S1: Low, high, SD and CI for the amino acids, Supplementary Results: stepwise-binomial logistic regression and Bootstrap resampling, Table S2: All statistical results.
