# Supplementary material for: Short-Term Effect of Induced Alterations in Testosterone Levels on Fasting Plasma Amino Acid Levels in Healthy Young Men
Source: Life (Basel). 2021 Nov 22;11(11):1276. doi: 10.3390/life11111276 (PMC8619397; doi:10.3390/life11111276)
Supplement: Supplementary file 1 [file life-11-01276-s001.zip › SahlinKB_supplementary/Supplementary Results - stepwise-binomial logistic regression and Bootstrap resampling.pdf]

Results from stepwise-binomial logistic regression (method: backward) and Bootstrap resampling with replacement method.

The model with the best results in the analysis included amino acids tyrosine and phenylalanine.

```
> library(MASS)
> mod_step <- stepAIC(mod1, direction = "backward", trace = F)
> mod_step

Call:  glm(formula = Time_point ~ Tyr + Phe, family = "binomial", data =
data2)

Coefficients:
(Intercept)          Tyr          Phe
    -95.398        3.444       12.763

Degrees of Freedom: 85 Total (i.e. Null);  83 Residual
Null Deviance:     109.9
Residual Deviance: 44.06       AIC: 50.06
> library(bootStepAIC)
> mod_boot <- boot.stepAIC(mod1, data = data2, B=50)
There were 50 or more warnings (use warnings() to see the first 50)
> mod_boot

Summary of Bootstrapping the 'stepAIC()' procedure for

Call:
glm(formula = Time_point ~ ., family = "binomial", data = data2)

Bootstrap samples: 50
Direction: backward
Penalty: 2 * df

Covariates selected
    (%)
Tyr  86
Phe  82
His  46
Lys  46
Trp  42
Met  34
Leu  26
Val  24
Asn  18

Coefficients Sign
     + (%)  - (%)
Phe 100.00   0.00
Tyr 100.00   0.00
Lys  95.65   4.35
Asn  77.78  22.22
Trp  71.43  28.57
Leu  46.15  53.85
Val  41.67  58.33
Met  29.41  70.59
His   0.00 100.00
```

```
Stat Significance
      (%)
Phe 68.29
Tyr 51.16
Trp 42.86
Met 41.18
Asn 33.33
Lys 30.43
Val 25.00
His 21.74
Leu  7.69
```

The stepAIC() for the original data-set gave

```
Call:  glm(formula = Time_point ~ Tyr + Phe, family = "binomial", data = 
data2)

Coefficients:
(Intercept)          Tyr          Phe  
    -95.398        3.444       12.763  

Degrees of Freedom: 85 Total (i.e. Null);  83 Residual
Null Deviance:     109.9 
Residual Deviance: 44.06      AIC: 50.06

Stepwise Model Path 
Analysis of Deviance Table

Initial Model:
Time_point ~ Asn + Val + Met + Leu + Tyr + Phe + Lys + His + 
    Trp

Final Model:
Time_point ~ Tyr + Phe


   Step Df   Deviance Resid. Df Resid. Dev      AIC
1                            76   39.96552 59.96552
2 - Leu  1 0.02320367        77   39.98872 57.98872
3 - Met  1 0.02213946        78   40.01086 56.01086
4 - Asn  1 0.06069513        79   40.07156 54.07156
5 - Val  1 0.10460726        80   40.17617 52.17617
6 - Trp  1 0.91316813        81   41.08933 51.08933
7 - Lys  1 1.60707345        82   42.69641 50.69641
8 - His  1 1.36787326        83   44.06428 50.06428
```
